# Immunoglobulin Abnormalities in Gaucher Disease: an Analysis of 278 Patients Included in the French Gaucher Disease Registry

**DOI:** 10.3390/ijms21041247

**Published:** 2020-02-13

**Authors:** Yann Nguyen, Jérôme Stirnemann, Florent Lautredoux, Bérengère Cador, Monia Bengherbia, Karima Yousfi, Dalil Hamroun, Leonardo Astudillo, Thierry Billette de Villemeur, Anaïs Brassier, Fabrice Camou, Florence Dalbies, Dries Dobbelaere, Francis Gaches, Vanessa Leguy-Seguin, Agathe Masseau, Yves-Marie Pers, Samia Pichard, Christine Serratrice, Marc G. Berger, Bruno Fantin, Nadia Belmatoug, on behalf of the French Evaluation of Gaucher Disease Treatment Committee

**Affiliations:** 1 Service de Médecine Interne, Centre de Référence des Maladies Lysosomales, AP-HP.Nord, Site Beaujon, Université de Paris, F-92110 Clichy, France; monia.bengherbia@aphp.fr (M.B.); karima.yousfi@aphp.fr (K.Y.); bruno.fantin@aphp.fr (B.F.); nadia.belmatoug@aphp.fr (N.B.); 2 Centre de Recherche en Epidémiologie et Santé des Populations, INSERM U1018, Université Paris-Sud, F-94805 Villejuif, France; 3 Service de Médecine Interne, Hôpitaux Universitaires de Genève, CH-1211 Geneva, Switzerland; jerome.stirnemann@hcuge.ch; 4 Service de Médecine Interne, CHU Pontchaillou, F-35000 Rennes, France; florent.lautredoux@chu-rennes.fr (F.L.); berengere.cador@chu-rennes.fr (B.C.); 5 Direction de la Recherche et de l’Innovation, CHRU de Montpellier, F-34295 Montpellier, France; d-hamroun@chu-montpellier.fr; 6 Service de Médecine Interne, CHU Toulouse, F-31300 Toulouse, France; leonardo.astudillo31@gmail.com; 7 Service de Neuropédiatrie, Hôpital Trousseau, Assistance Publique-Hôpitaux de Paris, F-75012 Paris, France; thierry.billette@aphp.fr; 8 Centre de Référence des Maladies Héréditaires du Métabolismes, Hôpital Necker-Enfants Malades, Assistance Publique-Hôpitaux de Paris, IHU Institut Imagine, F-75015 Paris, France; anais.brassier@aphp.fr; 9 Service de Réanimation Médicale, CHU Haut Levêque, F-33600 Pessac, France; fabrice.camou@chu-bordeaux.fr; 10 Institut de Cancéro-Hématologie, CHRU Morvan, F-29200 Brest, France; florence.dalbies@chu-brest.fr; 11 Centre de Référence des Maladies Héréditaires du Métabolisme de L’enfant et de L’adulte, CHRU de Lille, F-5900 Lille, France; dries.dobbelaere@chru-lille.fr; 12 Service de Médecine Interne, Hôpital Joseph Ducuing, F-31300 Toulouse, France; fgaches@hjd.asso.fr; 13 Service de Médecine Interne et Immunologie Clinique, CHU Bocage Central, F-21000 Dijon, France; vanessa.leguy-seguin@chu-dijon.fr; 14 Service de Médecine Interne, CHU Hôtel Dieu, F-44000 Nantes, France; agathe.masseau@chu-nantes.fr; 15 IRMB, Université de Montpellier, Inserm U1183, CHU Montpellier, F-34295 Montpellier, France; ympers2000@yahoo.fr; 16 Service des Maladies Métaboliques, Hôpital Robert Debré, F-75019 Paris, France; samia.pichard@aphp.fr; 17 Département de Médecine Interne de l’âgé, Hôpitaux Universitaires de Genève, CH-1226 Thonex, Switzerland; Christine.serratrice@hcuge.ch; 18 Service d’Hématologie Biologique et Service d’Hématologie Clinique Adulte, CHU Estaing, F-63000 Clermont-Ferrand, France; mberger@chu-clermontferrand.fr; 19 EA 7453 CHELTER, Université Clermont Auvergne, F-63000 Clermont-Ferrand, France

**Keywords:** Gaucher disease, polyclonal gammopathy, monoclonal gammopathy, multiple myeloma, monoclonal gammopathy of unknown significance

## Abstract

Gaucher disease (GD) is a rare lysosomal autosomal-recessive disorder due to deficiency of glucocerebrosidase; polyclonal gammopathy (PG) and/or monoclonal gammopathy (MG) can occur in this disease. We aimed to describe these immunoglobulin abnormalities in a large cohort of GD patients and to study the risk factors, clinical significance, and evolution. Data for patients enrolled in the French GD Registry were studied retrospectively. The risk factors of PG and/or MG developing and their association with clinical bone events and severe thrombocytopenia, two markers of GD severity, were assessed with multivariable Cox models and the effect of GD treatment on gammaglobulin levels with linear/logarithmic mixed models. Regression of MG and the occurrence of hematological malignancies were described. The 278 patients included (132 males, 47.5%) were followed up during a mean (SD) of 19 (14) years after GD diagnosis. PG occurred in 112/235 (47.7%) patients at GD diagnosis or during follow-up and MG in 59/187 (31.6%). Multivariable analysis retained age at GD diagnosis as the only independent risk factor for MG (> 30 vs. ≤30 years, HR 4.71, 95%CI [2.40–9.27]; *p* < 0.001). Risk of bone events or severe thrombocytopenia was not significantly associated with PG or MG. During follow-up, non-Hodgkin lymphoma developed in five patients and multiple myeloma in one. MG was observed in almost one third of patients with GD. Immunoglobulin abnormalities were not associated with the disease severity. However, prolonged surveillance of patients with GD is needed because hematologic malignancies may occur.

## 1. Introduction

Gaucher disease (GD, (OMIM #230800, #230900, #231000) is a rare lysosomal autosomal recessive disorder due to a deficiency of glucocerebrosidase (Enzyme Commission number EC 3.2.1.45), a lysosomal enzyme, or more rarely, its activator (saposin C) [1]. This deficiency leads to an accumulation of its substrate, glucosylceramide, in macrophages, which then accumulate in the bone marrow, liver, spleen, brain, and lungs. GD is one of the most common lysosomal disorders and is characterized by its heterogeneity, from an asymptomatic form to lethal forms. The main manifestations of GD include splenomegaly, hepatomegaly, bone involvement such as bone infarcts, avascular osteonecrosis, or pathological fractures, anemia, and/or thrombocytopenia. On the basis of neurological involvement, three distinct clinical phenotypes of GD have been described: type 1 GD is the most common phenotype (prevalence: 90–95% in Europe and North America) and typically causes no neurological damage; type 3 GD is characterized by neurological impairment, including horizontal ophtalmoplegia, myoclonus epilepsy, cerebellar ataxia, and/or dementia; type 2 GD is characterized by early severe neurological impairment, death occurring before the third year of life; the forms might represent a continuum [2]. GD is also associated with higher risks of some disease, such as Parkinson’s disease, solid cancer (i.e., hepatocellular carcinoma), and immunoglobulin abnormalities [1,3,4]. There are currently two types of treatment for GD: enzyme replacement therapy (ERT), supplying the GCase deficiency in the cells (imiglucerase, velaglucerase, or taliglucerase), and substrate reduction therapy (SRT), inhibiting GCase biosynthesis (miglustat or eliglustat) [1]. Their goal is to prevent complications such as massive splenomegaly, cytopenia, or avascular osteonecrosis, but they are not justified for all GD patients.

In the general population, polyclonal gammopathy (PG) occurs in many contexts, such as chronic inflammation, liver diseases, autoimmune diseases, infections, and malignancies and results in an overproduction of immunoglobulin by B lymphocytes. Monoclonal gammopathy (MG) is considered as a telltale sign of a B clonal proliferation, which can be benign or malignant. It can constitute premalignant states such as MG of unknown significance (MGUS) or in malignant B-cell hemopathy, such as multiple myeloma (MM), non-Hodgkin lymphoma (NHL), or chronic lymphocytic leukemia [5,6]. The prevalence of MGUS tends to increase with age and in particular from the age of 50 years, when it is estimated at 2%, and the risk of progression to MM is 1% per year [6].

In GD, immunoglobulin abnormalities are frequent, ranging from 21% to 91% for PG and 1% to 35% for MG according to previously published series [7,8,9,10,11,12,13,14,15,16,17]. These abnormalities can be present at GD diagnosis, sometimes leading to its diagnosis, and/or during follow-up. Moreover, the risk of MM is higher in GD patients than in the general population, with relative risk ranging from 1.3 to 51 [15,18,19]. 

The pathophysiology of these immunoglobulin abnormalities in GD is still unclear. Several factors have been hypothesized to play a role in their onset. Some authors suggested that the chronic inflammation state and an increase in levels of inflammatory cytokines such as interleukins (IL-6, IL-10) could lead to an overproduction of immunoglobulin [10]. More recently, Nair et al. showed that in GD, B lymphocytes were activated by specific type II natural killer T lymphocytes, with a T follicular helper profile, and the clonal immunoglobulin in GD patients and in mouse models of GD was reactive against glucosylsphingosine, the deacetylated form of glucosylceramide [20,21]. However, for Preuss et al., the target of paraprotein in GD-associated MGUS or MM was more likely saposin C, the glucocerebrosidase activator [22]. Despite debates on the pathophysiology, the risk factors, clinical significance and evolution of these gammopathies in GD are still unclear and have often been investigated in small cohorts. 

This study aimed to describe the immunoglobulin abnormalities PG and MG in a large cohort of GD patients and study the risk factors, clinical significance, and evolution with or without GD treatment.

## 2. Results

### 2.1. Study Population

Among the 657 GD patients included in the French GD registry (FGDR), 278 (42.3%) had at least one determination of immunoglobulin level and/or data on the presence or absence of MG and were included in the study (population 1). Most patients had type 1 GD (*n* = 262); three and 13 had type 2 and 3.

In total, 235/278 (84.5%) patients had at least one determination of immunoglobulin level (population 2), 187 (67.3%) had information on the presence or absence of MG (population 3), and 144 (51.8%) had both (population 4). Their main clinical demographic characteristics at GD diagnosis and during follow-up are in Table 1. Mean (standard derivation [SD]) age at GD diagnosis was 24.4 (18.3) years and mean follow-up duration since GD diagnosis was 19 (14) years.

### 2.2. Prevalence of PG and MG

**Figure A1:**
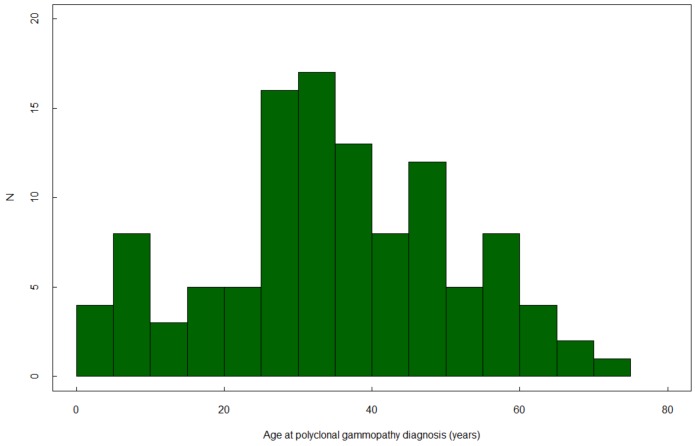
Age at polyclonal gammopathy diagnosis (*n* = 112).

Among the 235 patients with at least one determination of immunoglobulin level (population 2), 112 (47.7%) had PG at least once, either at GD diagnosis (*n* = 16; 14.3%) or during follow-up (*n* = 96; 85.7%) after a mean (SD) of 15 (11.2) years after diagnosis. The mean (SD) immunoglobulin level was 18.6 (5.3) g/L, with a maximum of 36 g/L. Mean age at PG diagnosis was 35.0 (16.5) years (Appendix A Figure A1), and mean duration between GD diagnosis and the first detection of PG was 12.9 (11.7) years.

**Figure A2:**
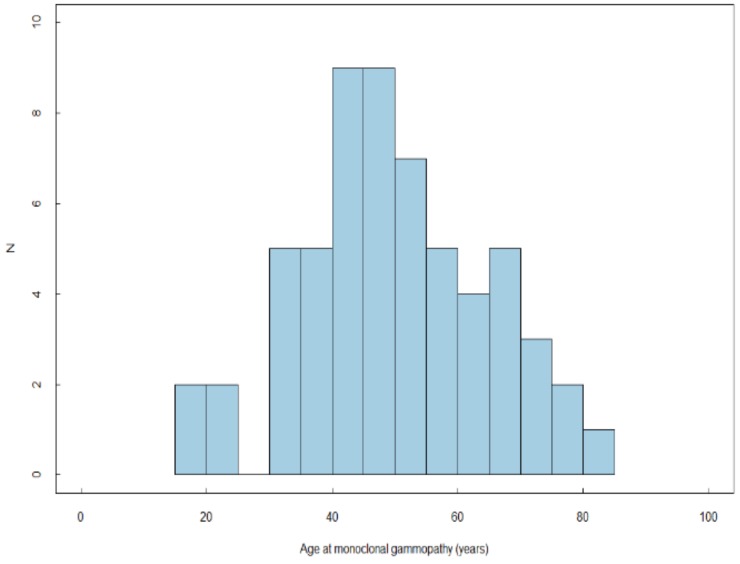
Age at monoclonal gammopathy diagnosis (*n* = 59).

Among the 187 patients with at least one determination of the presence or absence of MG (population 3), 59 (31.6%) had at least one recorded MG: 20 (10.7%) at GD diagnosis, and 39 (20.9%) during follow-up, after a mean (SD) of 19 (11) years after GD diagnosis. For these patients, mean age at GD diagnosis was 37.2 (18.5) years, and mean age at MG diagnosis was 49.7 (14.3) years (Appendix A Figure A2). All had type 1 GD. The type of the paraprotein was available for 50 (87.7%) patients: IgG-κ (*n* = 23), IgG-λ (*n* = 7), IgG with unknown type of light chain (*n* = 4), IgA-κ (*n* = 3), IgM-κ (*n* = 4), IgM-λ (*n* = 2), IgM with unknown type of light chain (*n* = 1), and free light chain λ (*n* = 1). In addition, four patients had biclonal gammopathy (IgG-κ and IgG-λ for 2, and IgM-λ and IgM-κ for 2).

### 2.3. Risk Factors of PG and MG

**Figure 1:**
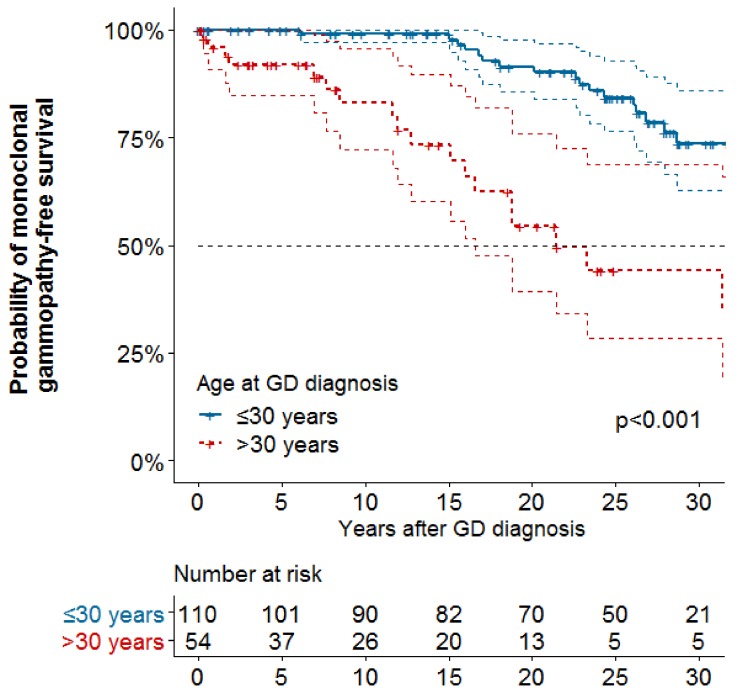
Kaplan-Meier curve of survival without monoclonal gammopathy by age at Gaucher disease diagnosis (*n* = 164). No: number; GD: Gaucher disease.

Associations between baseline characteristics, splenectomy and treatment and PG or MG are reported in Table 2. On univariate analyses, age at GD diagnosis was associated with increased risk of PG (hazard ratio [HR] 1.01; 95% confident interval [95%CI] 1.00–1.03) and MG (HR 1.08 [95%CI 1.05–1.1]). On multivariable analyses, no variables were associated with increased risk of PG, but age at GD diagnosis was associated with increased risk of MG (> 30 vs. ≤30 years: HR 4.71, 95%CI [2.40–9.27]) (Figure 1). Splenectomy and GD treatments did not decrease the risk of PG or MG on univariate and multivariable analyses.

On restricting our analysis to population 4, with available data on immunoglobulin level and MG, risk of MG was not increased with PG at diagnosis or during follow-up, assessed as a time-dependent variable (HR 0.83, 95%CI [0.36–1.90]), and risk of PG was not associated with MG at diagnosis or during follow-up (HR 0.81, 95%CI [0.34–1.91]).

### 2.4. Associations with Bone Events and Severe Thrombocytopenia

Associations between PG and MG with BE are shown in Table 3 and Table 4, respectively. Incident bone events (BE) (i.e., diagnosed after GD diagnosis) occurred for 76/190 (40.0%) and 61/150 (40.7%) patients included in the analyses for PG and MG after having excluded prevalent BE, respectively. On multivariable analyses, risk of BE was not significantly associated with presence of PG or but was significantly increased with splenectomy (HR 2.63, 95%CI [1.60–4.33] for PG; HR 2.60, 95%CI [1.49–4.55] for MG).

Incident severe thrombocytopenia (i.e., diagnosed after GD diagnosis) occurred for 47/197 (23.9%) and 36/151 (23.8%) patients included in the analyses for PG and MG, respectively. Similarly, risk of severe thrombocytopenia was not significantly associated with presence of PG or MG but was reduced with splenectomy or GD treatment ERT or SRT (Table 3 and Table 4). 

### 2.5. Evolution of PG and MG

**Figure 2:**
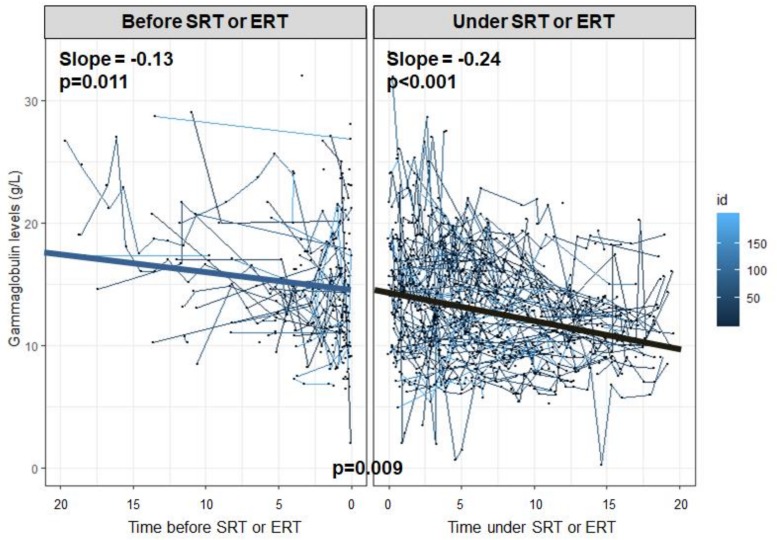
Evolution of gammaglobulin level before and after GD treatment. Treatment included enzyme replacement therapy or substrate reduction therapy. Slopes were obtained with linear/logarithmic mixed models on patients who received treatment, at two periods: before and after starting treatment. The impact of the treatment was assessed by using an interaction term between period and time.

Evolution of gammaglobulin levels as a function of time since the beginning of GD treatment (ERT or SRT) is reported in Figure 2. The gammaglobulin level decreased both before the initiation of GD treatment (slope = −0.13 [−0.22; −0.03]) and after (slope = −0.24 [−0.28; −0.20]), with a statistically significant interaction term between period and time (*p =* 0.009). These findings suggest a marked decline of gammaglobulin levels with ERT or SRT.

Among the 59 patients with MG, 10 never received GD treatment (mean [SD] age at GD diagnosis 42.1 [26.8] years); 28 received treatment before the first onset of MG (mean [SD] age at GD diagnosis 42.1 [26.8] years) and 21 after the first onset of MG (mean [SD] age at GD diagnosis 27.9 [15.3] years). Nine patients showed MG regression, including two without or before GD treatment and seven under GD treatment.

### 2.6. Malignant Hemopathies

Among the 278 included patients, malignant hemopathies occurred in six, including one with MM and five with NHL. Their characteristics, immunoglobulin abnormalities and age at diagnosis of the hemopathy are in Table 5. For four patients, MG was diagnosed before or concomitantly with the malignant hemopathy, including the only patient with IgG-λ MM. Five patients had died by the date of the analysis.

## 3. Discussion

With an analysis of a large cohort of GD patients included in the FGDR, our study highlights several points in understanding immunoglobulin abnormalities in GD. We found a high rate of the immunoglobulin abnormalities MG and PG, with almost half of our patients having PG at least once and almost one third having MG. Age at GD diagnosis was associated with increased risk of MG but not PG, but we did not find any other risk factors for these abnormalities. Additionally, presence of PG or MG was not associated with increased risk of BE or severe thrombocytopenia. Finally, even if immunoglobulin levels seemed to decrease with time and regression of MG is possible, malignant hemopathies such as MM or NHL may develop in some patients.

In our series, prevalence of PG (48%) and MG (32%) agree with those already published, from 21% to 91% and 1% to 35%, respectively, despite wide discrepancies among the reported series, mainly because of different study populations (children and/or adults) [17]. In our series, we confirmed that the prevalence of MG was higher than that found in the general population, ranging from 0.7 and 3.2% among people older than 50 years [23], and not explained by a patient selection bias (male/female ratio was balanced and the French cohort is more in line with a Caucasian population) [24]. However, the distribution of the different types of MG is equivalent to that described for the non-GD population.

As in non-GD population, age appeared to influence prevalence of MG [13,14]. In a series of 63 patients, de Fost et al. reported significantly older age for patients with than without MG (60 vs. 51 years; *p* = 0.003), and like our series, no other risk factors of MG were identified [13].

In our study, we did not find an association between PG and risk of MG, which does not agree with the mechanism proposed by Taddei et al., the suggested natural history being a temporal sequence of PG, followed by MG and eventually MM [15]. However, restricting the population to those with both data in our study might have decreased the power to find any association. Finally, starting ERT or SRT did not seem associated with decreased risk of the immunoglobulin abnormalities, but our analyses might have lack of power due to the small number of untreated patients. However, even if our study was not designed to study the effect of these treatments on PG or MG, our results do not suggest their efficacy in preventing these abnormalities. This finding is corroborated by the number of patients in whom PG and/or MG developed after ERT or SRT.

In our series, having an immunoglobulin abnormality did not seem associated with a more severe GD phenotype, here estimated by the occurrence of BEs or severe thrombocytopenia. We chose these two endpoints for their clinical relevance and because they reflect two different pathophysiological mechanisms. Our findings agree with previous studies. In a series of 228 patients, Brautbar et al. showed that the prevalence of BEs and splenectomies was not significantly increased with immunoglobulin abnormalities compared to without [11]. In their series, de Fost et al. showed that GD severity, estimated by the severity score index, and chitotriosidase levels, considered a reliable biomarker of GD, did not differ between patients with and without MG [13]. However, those findings relied on a comparison at one moment and were not assessed with Cox proportional-hazard models, taking temporality into account. These findings suggest that having an immunoglobulin abnormality does not seem a biomarker for assessing GD severity, and thus, the single presence of MG and/or PG should not be an indication to start ERT or SRT. Explaining this lack of correlation with GD has been difficult. Although glucosylsphingosine seem implicated in the pathogenesis and has been found a reliable biomarker of GD activity, the association between the level of glucosylsphingosine and risk of PG and/or MG is still unclear [25,26,27].

Immunoglobulin level seemed to decrease rapidly under ERT or SRT, although we could not perform statistical analyses because of the small number of events. In previous published series, results are divergent. In a series of 228 patients, Brautbar et al. observed a significant decrease in immunoglobulin level under treatment (mainly IgG), without a decrease of the monoclonal component [11]. However, MG regression under ERT was reported in some small series or case reports [14,28].

In our series, MM developed in only one patient after MG, and NHL developed in five patients. The risk of MM among GD patients varies widely but seems to be increased as compared with the general population (relative risk from 1.3 to 51.1), as is the risk of lymphoma (relative risk 2.5) [15,17,18,19]. Because of the limited number of hematological malignancies, we were not able to determine whether the risk of transformation from MGUS to MM was increased among GD patients nor assess the effect of ERT or SRT on risk of MM or NHL developing in GD patients. In addition, in at least two patients, the discovery of a PG at the time of diagnosis of the hemopathy suggests that the MG may be related to clonal lymphoid proliferation rather than GD. We lack sufficient data to recommend starting ERT or SRT in case of immunoglobulin abnormalities to prevent MM or NHL, but we recommend that these patients be monitored carefully.

We acknowledge some limitations to our study. First, our study was based on a national registry, supplied by data provided from different centers and clinicians. Even if many efforts are made to try to keep the registry as exhaustive as possible, there are many missing data, especially at GD diagnosis or for genotype. Thus, our analysis involved only 278 patients (42.3%) because of the lack of data for PG or MG, mainly followed in a GD referral center, which might suggest a selection bias. Our selection of patients for whom data on the presence or absence of MG was reported might have overestimated our reported prevalence of MG in our cohort. Moreover, biological data concerning these immunoglobulin abnormalities were not available at diagnosis for patients with a diagnosis when national guidelines on GD management were not yet published and when the risk of MM was underrecognized. This situation might imply bias by reducing the time of exposure to PG or MG, thus, weakening our findings. In addition, some data were missing regarding the type and rate of the monoclonal component or new criteria as light chain ratio, but further attempts are ongoing to complete our data. Finally, malignant hemopathies might have been underdeclared in our registry, which could bias our findings.

However, our study has several strengths. First, we included a large number of patients, with repeated visits during an extended follow-up of almost 20 years since GD diagnosis, followed in multiple centers providing real-life data. We also included patients with and without treatment. Moreover, most studies of immunoglobulin abnormalities in GD patients provided descriptive analyses or comparison of percentages, but to our knowledge, our study is the only one using Cox models, with time-dependent variables, providing a more precise evaluation of the exposure duration and a better consideration of confusion factors.

## 4. Materials and Methods

### 4.1. The French GD Registry

The FDGR was developed in 2009 by the Referral Center for Lysosomal Diseases and its Committee of Evaluation of Gaucher Disease Treatment [29]. Its objectives are to improve overall patient clinical care and professional practices and collect epidemiological data. The registry includes all GD patients followed in France since 1980. Clinical information and biological and bone findings at GD diagnosis and during follow-up are recorded, with the identification of intercurrent events, particularly bone complications or malignancies. The registry was approved by the French Data-Protection Commission and certified by the French Institute for Public Health Surveillance and the National Institute of Health and Medical Research (INSERM). All patients gave oral or written informed consent for use of their data.

### 4.2. Study Population

We conducted a retrospective multicentric study of data in the FGDR. For all patients, GD was diagnosed by the demonstration of deficient glucocerebrosidase activity in leukocytes. All GD patients with at least one determination of gammaglobulin level and/or with information on the presence or absence of MG (at baseline or during follow-up) were included in the study. We defined four populations: (1) the entire cohort (at least one determination of gammaglobulin level or presence or absence of MG), (2) the subgroup with at least one determination of gammaglobulin level, (3) the subgroup with at least one determination of the presence or absence of MG, (4) the subgroup with at least one determination of both gammaglobulin level and presence or absence of MG (patients were both in subgroups 2 and 3).

### 4.3. Baseline Characteristics and Collected Data

Baseline characteristics including age at GD diagnosis, socio-demographic characteristics, clinical and biologic findings, phenotypes and genotypes were extracted from the standardized record forms of the FGDR. Anemia was defined as hemoglobin level <10 g/dL, and thrombocytopenia as a platelet count <100 G/L. Because this was a retrospective study, some data were missing, particularly at GD diagnosis. If missing, biological data during the past or the next 2 years around the GD diagnosis were considered similar to those at diagnosis. GD treatment included ERT (imiglucerase, alglucerase, velaglucerase) or SRT (miglustat, eliglustat).

### 4.4. Outcomes

PG was defined by gammaglobulin level >15 g/L [30] and MG by the presence of paraprotein on serum protein electrophoresis and/or a monoclonal component with immunofixation. PG and MG data were collected at GD diagnosis, if available, and during follow-up.

To assess GD severity, two manifestations were collected: symptomatic bone events (BEs) and severe thrombocytopenia. BEs were defined by a bone infarct, a pathological fracture and/or an avascular osteonecrosis, with radiological confirmation. Severe thrombocytopenia was defined by platelet count <50 G/L. We chose these two endpoints because of their clinical relevance and because they reflect two different pathophysiological mechanisms.

Regression of the monoclonal component was defined by complete regression of a visible paraprotein on serum protein electrophoresis. Data on hematological malignancies (i.e., MM, lymphoma, leukemia) during follow-up were also collected.

### 4.5. Statistical Analysis

Baseline characteristics are described with mean (SD) or median (interquartile range [IQR]) for continuous variables and number (%) for categorical variables. Descriptive analysis was performed for each of the four populations. Survival functions were estimated by the Kaplan-Meier method. Factors associated with time-to event outcomes were evaluated with Cox proportional-hazards regression models, estimating HRs and 95%Cis. Time at entry in the models was the date of GD diagnosis, and exit time was the date of the event, death, or last visit, whatever occurred first.

To study risk factors of PG and MG, survival analyses were performed in populations 2 and 3, respectively, with the first PG and MG diagnosed used as events. Patients with prevalent PG and MG (i.e., diagnosed simultaneously with GD) were excluded from the analyses to study incident PG and MG (i.e., occurring after GD diagnosis). Studied variables included age, sex, genotype, phenotype, splenomegaly, hepatomegaly, anemia and thrombocytopenia at GD diagnosis. Additionally, splenectomy and GD treatment at baseline or during follow-up were assessed as time-dependent variables. To assess the association between PG and MG, survival analyses were performed for population 4, with data available for both. Variables with *p* < 0.2 on univariate analysis and splenectomy and GD treatment were used in the multivariable model.

Survival analyses were used to assess the association of PG and MG with GD severity, with the date of the first BE and the first severe thrombocytopenia used as events. To study incident BE and severe thrombocytopenia (i.e., occurring after GD diagnosis), patients with BE or severe thrombocytopenia at GD diagnosis were excluded from these analyses. PG and MG were modeled as time-dependent variables. Multivariable models were adjusted for variables associated with severe events at *p* < 0.2 on univariate analysis and splenectomy and GD treatment, modeled as time-dependent covariables.

The evolution of gammaglobulin levels and the impact of GD treatment were assessed by linear/logarithmic mixed models for patients who received treatment. Slopes were calculated for two periods—before and after starting ERT or SRT—and the impact of the treatment was assessed by using an interaction term between period (before or after GD treatment) and time. Regressions of the monoclonal component were described, as was the occurrence of malignant hemopathies.

Two-tailed *p* < 0.05 was considered statistically significant. All analyses were performed with R v3.6.2 (R Foundation for Statistical Computing, Vienna, Austria).

This study was performed in accordance with the ethical standards of the Helsinki Declaration and was approved by the local Institutional Review Board of Assistance Publique-Hôpitaux de Paris Nord, Paris University (IRB00006466).

## 5. Conclusions

Immunoglobulin abnormalities are more frequent in GD but are not associated with a severe GD phenotype. ERT or SRT should not be started based solely on the presence of these abnormalities. These patients should be monitored with caution regarding hematological malignancies, at least by respecting the usual guidelines for MGUS follow-up. Efforts are still needed to better understand the pathophysiology of these abnormalities in GD patients and the influence of specific treatment.

## Figures and Tables

**Table 1:** Baseline characteristics of participants with Gaucher disease (GD) with or without polyclonal gammopathy (PG) or monoclonal gammopathy (MG).

	Population 1 (*n* = 278) *(All Cohort)*	Population 2 (*n* = 235)		Population 3 (*n* = 187) *(At Least One Determination of Presence or Absence of MG)*	
		*(At Least One Gammaglobulin Determination)*			
		PG–	PG+	MG–	MG+
**Characteristics at GD diagnosis**	*n* = 278	*n* = 123	*n* = 112	*n* = 128	*n* = 59
Sex = male, *n* (%)	132 (47.5)	62 (50.4)	47 (42.0)	59 (46.1)	31 (52.5)
Age at GD diagnosis, *years*, *n* (%)	24.4 (18.3)	24.1 (19.8)	22.0 (15.2)	22.6 (15.2)	37.2 (18.5)
>30 years old	91 (32.7)	43 (35.0)	28 (25.0)	37 (28.9)	36 (61.0)
Phenotype, *n* (%)					
type 1 GD	262 (94.2)	113 (91.9)	106 (94.6)	123 (96.1)	59 (100)
type 2 GD	3 (1.1)	3 (2.4)	0 (0.0)	0 (0.0)	0 (0.0)
type 3 GD	13 (4.7)	7 (5.7)	6 (5.4)	5 (3.9)	0 (0.0)
Genotype, *n* (%)					
*p.Asn409Ser/p.Asn409Ser*	29 (10.4)	14 (11.4)	15 (13.4)	18 (14.1)	6 (10.2)
*p.Asn409Ser/p.Leu483Pro*	45 (16.2)	15 (12.2)	20 (17.9)	26 (20.3)	9 (15.3)
*p.Leu483Pro/p.Leu483Pro*	5 (1.8)	2 (1.6)	3 (2.7)	2 (1.6)	0 (0.0)
*p.Asn409Ser*/other	68 (24.5)	41 (33.3)	21 (18.8)	31 (24.2)	16 (27.1)
*p.Leu483Pro*/other	9 (3.2)	4 (3.3)	3 (2.7)	6 (4.7)	1 (1.7)
NA	122 (43.9)	47 (38.2)	50 (44.6)	45 (35.2)	27 (45.8)
Splenomegaly at GD diagnosis, *n* (%)					
No	58 (20.9)	11 (8.9)	9 (8.0)	11 (8.6)	3 (5.1)
Yes	129 (46.4)	89 (72.4)	79 (70.5)	91 (71.1)	35 (59.3)
NA	64 (23.0)	23 (18.7)	24 (21.4)	26 (20.3)	21 (35.6)
Hepatomegaly at GD diagnosis, *n* (%)					
No	58 (20.9)	30 (24.4)	18 (16.1)	34 (26.6)	7 (11.9)
Yes	129 (46.4)	58 (47.2)	57 (50.9)	57 (44.5)	25 (42.4)
NA	91 (32.7)	35 (28.5)	37 (33.0)	37 (28.9)	27 (45.8)
Anemia at GD diagnosis, *n* (%)					
No	131 (47.1)	73 (59.3)	39 (34.8)	68 (53.1)	28 (47.5)
Yes	37 (13.3)	12 (9.8)	20 (17.9)	15 (11.7)	6 (10.2)
NA	110 (39.6)	38 (30.9)	53 (47.3)	45 (35.2)	25 (42.4)
Thrombocytopenia at diagnosis, *n* (%)					
No	84 (30.2)	43 (35.0)	26 (23.2)	44 (34.4)	14 (23.7)
Yes	105 (37.8)	51 (41.5)	39 (34.8)	47 (36.7)	24 (40.7)
NA	89 (32.0)	29 (23.6)	47 (42.0)	37 (28.9)	21 (35.6)
**Characteristics during follow-up,**					
Splenectomy, *n* (%)	69 (24.8)	15 (12.2)	42 (37.5)	24 (18.8)	22 (37.3)
ERT/SRT, *n* (%)	228 (82.0)	101 (82.1)	97 (86.6)	102 (79.7)	49 (83.1)

Results are expressed are number (%) for categorical variables. GD: Gaucher disease; NA: not available; ERT: enzyme replacement therapy; SRT: substrate reduction therapy.

**Table 2:** Cox proportional-hazards analysis of risk of PG or MG with GD.

	Risk of PG								Risk of MG							
			Univariate Analysis			Multivariable Analysis					Univariate Analysis			Multivariable Analysis		
	*n*	PG	HR	95%CI	*p*	HR	95%CI	*p*	*n*	MG	HR	95%CI	*p*	HR	95%CI	*p*
Age at GD diagnosis, *years*	209	97	1.01	1.00–1.03	0.034	1.01	0.99–1.03	0.356	164	39	1.08	1.05–1.10	<0.001	1.08	1.05–1.10	<0.001
Age at GD diagnosis	209	97							164	39						
≤30 years			(ref)								(ref)					
>30 years			1.48	0.92–2.39	0.107						4.71	2.40–9.27	<0.001			
Male	209	97	0.92	0.61–1.39	0.69						1.3	0.69–2.46	0.414			
Genotype	126	52							104	21						
*p.Asn409Ser/p.Asn409Ser*			(ref)								(ref)					
*p.Leu483Pro/p.Leu483Pro*			1.15	0.25–5.17	0.86						-	-	-			
*p.Asn409Ser/p.Leu483Pro*			0.90	0.42–1.95	0.797						0.59	0.14–2.53	0.48			
Other			0.71	0.20–2.56	0.605						1.11	0.39–3.17	0.84			
Splenomegaly at diagnosis	164	73	0.66	0.30–1.45	0.302				123	35	1.06	0.14–8.02	0.954			
Hepatomegaly at diagnosis	138	60	1.04	0.56–1.92	0.91				106	18	2.58	0.59–11.27	0.207			
Anemia at diagnosis	120	45	1.76	0.95–3.26	0.071	1.82	0.95–3.51	0.072	101	20	0.69	0.23–2.07	0.506			
Thrombocytopenia at diagnosis	135	51	1.03	0.58–1.83	0.92				110	21	1.43	0.55–3.69	0.463			
Treatment during follow-up *^§^	209	97	0.89	0.56–1.43	0.635	0.80	0.40–1.62	0.536	164	39	1.35	0.67–2.73	0.405	1.25	0.61–2.60	0.543
Splenectomy during follow-up ^§^	209	97	1.17	0.76–1.82	0.471	1.22	0.57–2.61	0.602	164	39	1.04	0.52–2.05	0.917	0.98	0.49–1.98	0.963
MG ^§^	135	63	0.81	0.34–1.91	0.625											
PG ^§^									135	28	0.83	0.36–1.90	0.652			

Univariate analyses involved the log rank test. Survival was calculated from the time of GD diagnosis to the first occurrence of PG, MG, or the last follow-up visit. Multivariable analyses involved 120 patients (45 events) for PG and 164 patients (39 events) for MG and included variables with *p* < 0.2 in univariate analyses, treatment and splenectomy. * Treatment included enzyme replacement therapy or substrate reduction therapy. ^§^ at diagnosis or during follow-up (time-dependent variables). HR: hazard ratios; 95%CI: 95% confidence interval; GD: Gaucher disease; PG: polyclonal gammopathy; MG: monoclonal gammopathy; ref: reference.

**Table 3:** Risk of bone events and severe thrombocytopenia with PG.

	Risk of Bone Events								Risk of Severe Thrombocytopenia							
			Univariate Analysis			Multivariable Analysis					Univariate Analysis			Multivariable Analysis		
	*n*	Events	HR	95%CI	*p*	HR	95%CI	*p*	*n*	Events	HR	95%CI	*p*	HR	95%CI	*p*
**PG** ^§^	190	76	1.29	0.73–2.27	0.381	1.27	0.70–2.30	0.427	197	47	0.89	0.42–1.91	0.769	1.19	0.55–2.59	0.658
Age at GD diagnosis	190	76	1.01	0.99–1.02	0.476	1.01	0.99–1.02	0.503	197	47	1.01	0.99–1.03	0.209	1.01	0.99–1.03	0.296
Male sex	190	76	0.95	0.59–1.51	0.818	1.25	0.76–2.06	0.378	197	47	1.13	0.64–2.00	0.678	1.03	0.58–1.85	0.915
Splenectomy during follow-up ^§^	190	76	2.37	1.49–3.77	<0.001	2.63	1.60–4.33	<0.001	197	47	0.41	0.18–0.90	0.026	0.34	0.15–0.76	0.009
Treatment during follow-up *^§^	190	76	0.98	0.59–1.65	0.948	1.14	0.65–1.99	0.64	197	47	0.33	0.14–0.82	0.016	0.26	0.10–0.68	0.006

Survival was calculated from the time of GD diagnosis to the first occurrence of bone events or severe thrombocytopenia or the last follow-up visit. GD: Gaucher disease; PG: polyclonal gammopathy; HR: hazard ratios; 95%CI: 95% confidence interval; * Treatment included enzyme replacement therapy or substrate reduction therapy. ^§^ Time-dependent variables.

**Table 4:** Risk of bone events and severe thrombocytopenia with MG.

	Risk of Bone Events								Risk of Severe Thrombocytopenia							
			Univariate Analysis			Multivariable Analysis					Univariate Analysis			Multivariable Analysis		
	*n*	Events	HR	95%CI	*p*	HR	95%CI	*p*	*n*	Events	HR	95%CI	*p*	HR	95%CI	*p*
**MG** ^§^	150	61	1.40	0.69–2.86	0.353	1.28	0.60–2.75	0.525	151	36	0.58	0.14–2.42	0.453	0.55	0.12–2.47	0.439
Age at GD diagnosis	150	61	1.01	1.00–1.03	0.097	1.01	0.99–1.03	0.326	151	36	1	0.98–1.02	0.917	1.01	0.98–1.03	0.666
Male sex	150	61	1.04	0.62–1.73	0.713	1.28	0.73–2.23	0.525	151	36	0.96	0.50–1.86	0.906	0.81	0.41–1.60	0.548
Splenectomy during follow-up ^§^	150	61	**2.30**	**1.37–3.87**	**0.002**	**2.60**	**1.49–4.55**	**0.001**	151	36	0.53	0.22–1.25	0.148	0.46	0.19–1.11	0.085
Treatment during follow-up *^§^	150	61	1.22	0.70–2.14	0.479	1.44	0.81–2.57	0.218	151	36	0.6	0.24–1.46	0.261	0.56	0.23–1.38	0.207

Survival was calculated from the time of GD diagnosis to the first occurrence of bone events or severe thrombocytopenia, or the last follow-up visit. GD: Gaucher disease; MG: monoclonal gammopathy; HR: hazard ratios; 95%CI: 95% confidence interval; * Treatment included enzyme replacement therapy or substrate reduction therapy. ^§^ Time-dependent variables.

**Table 5:** Characteristics of the six patients with malignant hemopathy.

Patient	Sex	Age at GD Diagnosis (Years)	PG (g/L)	Age at PG Diagnosis (Years)	MG (type)	Age at First GM (years)	Malignant Hemopathy	Age at Hemopathy	Age at GD Treatment	Status
1	F	61	21.7	68	-	-	AITL	83	79	Deceased
2	F	29	25	47	-	-	B-NHL	57	45	Deceased
3	F	24	16.1	49	IgM λ	48	MALT lymphoma	47	51	Alive
4	F	56	22.1	61	IgG κ	65	Lymphocytic lymphoma	61	Not treated	Alive
5	M	42	-	-	IgG λ	42	B-NHL	54	NA	Deceased
6	F	62	-	-	IgG λ	75	MM IgG λ	75	75	Deceased

GD: Gaucher disease; PG: polyclonal gammopathy (immunoglobulin level); MG: monoclonal gammopathy; F: female; M: male; AITL: angioimmunoblastic T-cell lymphoma; MALT: mucosa-associated lymphoid tissue; NHL: non-Hodgkin lymphoma; MM: multiple myeloma; NA: not available.

## Abbreviations

GD	Gaucher disease
PG	polyclonal gammopathy
MG	monoclonal gammopathy
MGUS	monoclonal gammopathy of unknown significance
MM	multiple myeloma
NHL	non-Hodgkin lymphoma
FGDR	French Gaucher disease registry
SD	standard derivation
IQR	interquartile range
HR	hazard ratio
CI	confident interval
ERT	enzyme replacement therapy
SRT	substrate reduction therapy
